# Transcriptomic and regulatory landscape of liver tissue associated with meat and carcass quality traits in cattle

**DOI:** 10.1186/s40104-026-01465-0

**Published:** 2026-08-01

**Authors:** Thais Ribeiro da Silva, Juliana Afonso, Bárbara Silva-Vignato, Ingrid Soares Garcia, Beatriz Delcarme Lima, Heidge Fukumasu, Saulo Luz Silva, Juliana Petrini, Carolina Purcell Goes, Bruna Petry, Wellison J. S. Diniz, Aline Silva Mello Cesar, Gerson Barreto Mourão, James E. Koltes, Luciana Correia de Almeida Regitano, Luiz Lehmann Coutinho

**Affiliations:** 1 Department of Animal Science, College of Agriculture “Luiz de Queiroz”, University of São Paulo, Piracicaba, SP Brazil; 2 Embrapa Pecuária Sudeste, São Carlos, SP Brazil; 3 College of Animal Science and Food Engineering, University of São Paulo, Pirassununga, SP Brazil; 4 Department of Structural Biology, Federal University of Triângulo Mineiro, Uberaba, MG Brazil; 5 Department of Animal Science, Iowa State University, Ames, IA USA; 6 Department of Animal Science, Auburn University, Auburn, AL USA

**Keywords:** Carcass quality, Cattle, Chromatin accessibility, EQTL mapping, Multi-omics integration, WGCNA

## Abstract

**Background:**

The integration of multiple omics strategies represents a transformative paradigm in farm animal genetics and breeding. By capturing molecular complexity across biological layers, integrative omics offers new opportunities to reveal the regulatory mechanisms underlying economically important traits. Here, we characterize the hepatic transcriptomic landscape of Nelore cattle and its relationship with meat and carcass quality phenotypes. For that, we integrated gene expression data, co-expression networks, expression quantitative trait locus (eQTL) mapping, single-nucleotide polymorphism (SNP)–phenotype associations, chromatin accessibility, and transcription factor motif analyses using hepatic RNA-seq data from 90 animals and assay for transposase‑accessible chromatin using sequencing (ATAC-seq) data from two animals.

**Results:**

Weighted gene co-expression network analysis (WGCNA) identified 10 gene modules associated with our phenotypes of interest, particularly the blue module (*r* = −0.4), which is linked to meat color and enriched in insulin and mTOR signaling pathways. eQTL mapping revealed 1,198 *cis*- and 39,227 *trans-*eQTLs (false discovery rate [FDR] < 0.05), including hotspots on chromosome 25. Notably, rs449155362 was found to regulate 848 genes, within them *MLXIPL*, a transcription factor involved in glucose and lipid metabolism. Phenotype–eQTL associations revealed 54 SNPs (FDR < 0.05) related to meat and carcass traits, among which rs110069409, within an open chromatin region, modulates *PLA2G2D1* expression and was associated with meat color (yellowness 24 h after the slaughter—b*₀), representing a convergence point across regulatory layers.

**Conclusion:**

These findings provide novel insights into the multilayered genetic architecture of the liver that controls meat quality traits in beef cattle, supporting the use of integrative omics to guide functional genomic selection.

**Supplementary Information:**

The online version contains supplementary material available at 10.1186/s40104-026-01465-0.

## Background

Brazilian beef cattle production is predominantly based on Zebu breeds, especially Nelore, due to their adaptability to tropical climates. However, Nelore cattle typically produce meat with lower marbling and tenderness scores compared to European breeds, which poses challenges for consumer acceptance and market competitiveness [1]. Improving traits related to meat and carcass quality, such as tenderness, color, and fat deposition, is therefore a priority in genetic improvement programs.

A significant knowledge gap persists regarding the molecular basis of these economically important traits, particularly in Zebu breeds. Previous studies have identified candidate genes and quantitative trait loci (QTLs) associated with meat quality, but the regulatory mechanisms that control gene expression and influence phenotypic variation remain largely unknown. Understanding these mechanisms is essential for creating more precise selection strategies in breeding programs [2].

Advances in transcriptomics, particularly RNA sequencing (RNA-Seq), have enabled high-resolution profiling of gene expression, allowing researchers to investigate the molecular basis of complex traits. The transcriptome reflects the complete set of RNA molecules expressed in a tissue, providing insights into gene activity under specific physiological conditions [3]. When combined with systems biology approaches such as weighted gene co-expression network analysis (WGCNA), RNA-Seq data can reveal gene modules associated with phenotypes and uncover key regulators and biological pathways [4, 5].

In addition to gene expression profiling, RNA-Seq enables the identification of single-nucleotide polymorphisms (SNPs), which can be used to map expression quantitative trait loci (eQTLs), genetic variants that influence gene expression levels. Some eQTLs, termed “hotspots”, modulate the expression of many genes and may serve as central nodes in gene regulatory networks [6, 7]. To understand how such regulatory variants exert their effects, it is essential to investigate the chromatin landscape. Techniques like assay for transposase-accessible chromatin using sequencing (ATAC-seq) allow researchers to identify regions of open chromatin, which are typically associated with active regulatory elements such as promoters and enhancers. [8, 9]. These regions are crucial for transcription factor binding and gene regulation.

Variants located within regulatory regions can alter transcription factor binding affinity, thereby modulating gene expression and ultimately influencing phenotypic traits [10]. The annotation of these regions using chromatin state databases, such as those provided by the Functional Annotation of Animal Genomes (FAANG) consortium, enhances our understanding of the functional impact of genetic variation [11]. The integration of these multi-layered datasets represents a transformative approach in farm animal genetics. By capturing molecular complexity across different regulatory levels, integrative omics enables the discovery of mechanisms underlying economically important traits. Studies have further demonstrated the value of integrative multi-omics approaches for uncovering regulatory variants affecting gene expression and complex traits in livestock [12, 13], reinforcing the importance of combining transcriptomic and regulatory data to better understand phenotypic variation.

Here, we hypothesize that integrating multi-omics data from liver tissue can uncover key regulatory elements and networks associated with meat and carcass quality traits in Nelore cattle. This study aims to address a critical gap in understanding the molecular basis of these traits in Zebu breeds. To achieve this, we combine gene expression profiling, co-expression network analysis, eQTL mapping, SNP-phenotype associations, chromatin accessibility data, and transcription factor motif analysis.

## Materials and methods

### Animal management

Data were collected from an experimental herd at the Faculty of Animal Science and Food Engineering, University of São Paulo (FZEA/USP – Pirassununga, SP). The herd consisted of 90 uncastrated Nelore males, all raised on pasture in a continuous grazing system and finished in a feedlot for approximately 98 d as described in a previous study [14]. At the end of the feedlot period, the animals were slaughtered at the experimental slaughterhouse of USP, located about 300 m from the feedlot facilities. The animals had an average body weight of 578 ± 50 kg and an average age of 727 ± 50 d, in compliance with Brazilian legislation requirements.

### Ethics

The Faculty of Animal Science and Food Engineering Ethics Committee at the University of São Paulo (FZEA–USP) approved all procedures involving animals in this experiment (protocol number 8886050916/16).

### Sample collection and evaluated traits

Immediately after eviscerating the animals, liver samples were collected from 90 animals, preserved in liquid nitrogen, and stored in an ultra-freezer at −80 °C until further processing. During the slaughter process, each carcass was individually identified and weighed to assess quality-related parameters, including carcass weight (CW) and dressing percentage (DP). The carcasses were then split into two halves and chilled at 2 °C for 24 h.

After 24 h, cold carcass weight (CCW) was recorded, and the pH was measured in the *Longissimus* muscle between the 12^th^ and 13^th^ ribs. During the slaughter process, the ribeye area (cm^2^) and backfat thickness (mm) were measured on the *Longissimus* muscle cut (rib-eye steak) using a point grid and a graduated ruler, respectively. Subsequently, three 2.5 cm wide steak samples were extracted between the 11^th^ and 13^th^ ribs of the *Longissimus* muscle. Each steak was individually vacuum-packed and aged in a cold chamber at 2 °C for 0 d (immediately after slaughter), 7 d, or 14 d.

After each maturation period: 0 d (24 h postmortem), 7 d, and 14 d after slaughter, meat samples were evaluated for pH, color (L*, a*, b* system), cooking losses (CL) [15], and Warner–Bratzler shear force (SF) [16] for all samples.

### RNA extraction and sequencing

Total liver RNA was extracted from 100 mg of tissue using Trizol reagent (Life Technologies), following the manufacturer’s instructions. For quality control, the samples were evaluated by electrophoresis on a 1% agarose gel stained with GelRed. RNA purity was measured using the NanoDrop 2000 spectrophotometer, and concentration was measured using the Qubit (Thermo Fisher). Additionally, the RNA integrity was evaluated using the Bioanalyzer 2100 (Agilent Technologies). Samples with an RNA integrity number (RIN) greater than or equal to 7 were used for library preparation. To prepare the complementary DNA (cDNA) libraries, 2 µg of RNA from each sample was used, following the protocol described in the TruSeq RNA Sample Preparation kit v2 guide (Illumina).

Library quantification was determined by quantitative PCR, and library size was measured on the Bioanalyzer 2100. The libraries were then sequenced on the NextSeq 2000 (Illumina). Sample preparation and sequencing were performed at the Multi-User Laboratory of Functional Genomics Applied to Agriculture and Agroenergy, at ESALQ-USP. After sequencing, adapters and low-quality sequences were removed using the dFASTX-Toolkit tool v.0.0.13 (http://hannonlab.cshl.edu/fastx_toolkit/index.html). Alignment of reads against the *Bos taurus* ARS-UCD1.2 bovine reference genome was performed using the STAR v. 2.7 aligner (Spliced Transcripts Alignment to a Reference) [17]. Only reads that were uniquely mapped to autosome chromosomes were used in this study. To count the reads, we used the QuantMode function from STAR [17].

### Gene expression quantification

Gene-level expression quantification was obtained directly during alignment using the QuantMode option implemented in STAR v.2.7 [17], which generates raw read counts per gene based on the provided NCBI RefSeq gene annotation mapped to the *Bos taurus* ARS-UCD1.2 reference genome. The resulting raw count matrix across all samples was used as input for downstream analyses. Reads were filtered by removing genes with expression levels below 1 count in at least 50% of samples (45 animals). Subsequently, the filtered reads were normalized to counts per million (CPM) and transformed into log2 (log_2_CPM) using the edgeR package in R v.4.6.3 [18].

### SNP calling of the RNA-seq data

SNP calling was performed using GATK v4.1.0.0 following RNA-seq-specific best practices [19]. STAR-aligned reads were sorted by genomic coordinates, read groups were assigned, and reads containing Ns in their CIGAR strings were split using SplitNCigarReads prior to base quality score recalibration using known *Bos taurus* variants from Ensembl (release 95, January 2019) [20]. Variant calling was performed per sample using the *HaplotypeCaller* tool in Genomic Variant Call Format (GVCF) mode, followed by joint genotyping across all samples. Only high-quality biallelic autosomal SNPs meeting filtering identified criteria (QUAL > 30, depth > 10, call rate ≥ 95%, and minor allele frequency (MAF) ≥ 5%) were used herein. The SNPs were annotated using the Variant Effect Predictor (VEP) tool from Ensembl genome browser [21].

### Population stratification test

To assess population stratification, principal component analysis (PCA) was performed using SNP genotypes derived from RNA‑seq data for 90 animals with PLINK software [22]. Sire information was used only for visualization purposes to evaluate sample clustering patterns.

### Phenotype correction

Phenotypic variables were evaluated prior to association analyses using the PROC MIXED procedure in SAS to identify relevant fixed effects. The contemporary group (CG) was defined by slaughter batch and days on feedlot. Age and the first two genotype principal components (PC1 and PC2), calculated from RNA-seq–derived SNPs to account for population stratification (Fig. S1), were also fitted as covariates. Residuals from the final linear mixed-effects model were used exclusively for downstream WGCNA and SNP-phenotype association analyses.

### Weighted correlation network analysis (WGCNA)

For the co-expression network analysis, we utilized the WGCNA package (v.1.73) implemented in R [4]. Before network construction, additional filtering was applied to remove outlier samples and genes with excessive missing or zero values. Outlier samples were identified by hierarchical clustering of the sample dendrogram. Additionally, sample connectivity was calculated within the WGCNA framework, and samples with a connectivity Z-score below −2 (i.e., > 3 standard deviations from the mean sample connectivity) were excluded. Genes were filtered out if more than 20% of their expression values were missing or equal to zero across samples.

Modules were built using the signed network approach in WGCNA, which preserves the directionality of gene correlations by distinguishing positive from negative relationships. Modules were grouped if their eigengene correlation exceeded 0.75. Each gene module was assigned to a random specific color. To identify hub and transcription factor genes potentially regulating the phenotypes under investigation, we performed module-trait association analysis. To this end, module-trait associations were estimated by correlating the eigengene value of each module with the target trait [4].

### Functional enrichment analysis

We performed functional over-representation analyses to pinpoint the biological processes and pathways underlying the associated modules. Functional enrichment of the modules was performed using ShinyGO v0.61 [23]. Significance was defined as false discovery rate (FDR) < 0.1, and the background was defined as our set of genes expressed in the liver.

### Gene expression–phenotype association analysis

In addition to WGCNA, an association analysis was performed. This analysis aimed to identify variations in expression levels and how these variations impact the phenotype. It served as a secondary confirmation of the relationship between the genes and the phenotypes.

To investigate the association between gene expression levels and phenotypic variation, an association analysis was performed using the DESeq2 R package v.3.21 [24]. The raw RNA-Seq count data were first imported into R, and samples with missing values were excluded. For the phenotype, we used the residuals of the corrected traits. This approach fits a generalized linear model (GLM) with a negative binomial distribution. A DESeq2 object was then constructed using the *DESeqDataSetFromMatrix* function, where the design formula (~ phenotype) was defined to model gene expression as a function of phenotypic variation. This analysis was performed separately for each phenotype. According to Love et al. [24], when using a continuous variable in the design formula, the log_2_ fold change represents the effect of a unit increase in that variable on the log2-scaled counts.

The DESeq function fits the model and identifies genes whose expression levels are significantly associated with the phenotypic variable. Adjusted *P* values were calculated for each gene. Genes with adjusted *P* values (*P*_adj_) below 0.05 were significantly associated with the phenotype.

### eQTL analysis

The eQTL analysis was performed using the MatrixEQTL package v.2.3 in R [25] with a fixed linear model. SNPs were identified from RNA-seq data. For the eQTL analysis, we used genes from the ten significant modules identified by WGCNA and those associated with phenotypes, totaling 11,578 genes.

To account for potential confounding effects, the eQTL model included slaughter batch as a categorical covariate to control for technical and environmental batch effects, as well as the first two genotype principal components (PC1 and PC2) calculated from RNA-seq–derived SNP genotypes to correct for population structure. Separate tests were conducted with MatrixEQTL for each gene–SNP pair, with multiple-testing correction performed using the FDR method. *Cis-*eQTLs were defined as SNPs located within 1 Mb of the gene transcription start or end site, whereas *trans-*eQTLs were defined as variants located outside this region [25].

### Permutation test to identify eQTL hotspots

The term "eQTL hotspots" refers to regions that simultaneously impact the expression levels of multiple genes [6]. To identify these hotspots and determine which eQTLs regulate the highest number of genes, a permutation test was performed with 10,000 iterations, and eQTLs with a *P* < 0.05 were considered significant.

### Linkage disequilibrium analysis

Linkage disequilibrium (LD) was estimated using PLINK v1.9 [22]. The genotype data were converted to PLINK binary format, after which LD matrices were computed for all SNP pairs within each chromosome, with a maximum window size of 200 kb between adjacent markers. The LD window was defined as SNP pairs with a correlation (*r*^2^) of 0.8 or higher, allowing us to estimate the size of LD blocks.

### SNP-trait association

SNP-phenotype analyses were restricted to variants previously identified as eQTLs. This strategy reduced the multiple-testing burden while prioritizing variants with evidence of regulatory effects on gene expression, consistent with the study's exploratory and integrative scope. Associations between eQTL SNPs and phenotypes were tested using a linear model implemented in PLINK [22], with analyses conducted on phenotypic residuals from the phenotype correction step. Multiple testing correction was applied with an FDR < 0.05 [26].

### ATAC-seq

For the ATAC-seq assay, we utilized ~10 mg of frozen liver from two samples from Nelore males, purchased from a commercial slaughterhouse, replicated in two technical replicates for each, and submitted to a modified version of the OMNI-ATAC-seq method [27]. Briefly, the samples were minced and homogenized with 10 strokes using pestle A, followed by 20 strokes using pestle B. The samples were centrifuged at 100 × *g* for 1 min at 4 °C and resuspended in lysis buffer (10 mmol/L Tris–HCl, pH 7.4, 10 mmol/L NaCl, 3 mmol/L MgCl_2_, and 0.1% Tween-20). The nuclei were isolated using a 1:1:1 gradient of 27% Percoll, 2.5 mol/L sucrose, and the homogenate was centrifuged in a swinging-bucket centrifuge for 40 min at 3,000 × *g* at 4 °C. Tagmentation of 50,000 nuclei was performed using 2.5 µL Tn5 transposase in TM buffer (20 mmol/L Tris–HCl, pH 7.6, 10 mmol/L MgCl₂, 20% Dimethyl Formamide, 0.1% Tween-20, 0.01% Digitonin, 1 × PBS) and incubating at 37 °C for 40 min. Libraries were constructed using the Nextera DNA Library Prep Kit (Illumina, 15028212). Quality control of prepared libraries was performed using an Agilent 2100 Bioanalyzer for fragment analysis. Libraries were pooled to equimolar concentrations using the KAPA Library Quant Kit (cat# KK4854) and sequenced with paired-end 2 × 100 reads on an Illumina NextSeq2000 instrument.

Paired-end sequencing reads were trimmed using CutAdapt [28] and aligned to the *Bos taurus* bta9 reference genome using Bowtie 2 [29]. Genomic coordinates and gene annotations were based on the NCBI RefSeq database, mapped to the *bosTau9* assembly (https://www.ncbi.nlm.nih.gov/assembly/313678/). Duplicate reads were marked using Picard *MarkDuplicates*, and BAM files were filtered with SAMtools to discard unmapped reads, non-primary alignments, reads failing platform/vendor quality checks, and PCR or optical duplicates (-f 2 -F 1804). Quality metrics were assessed using ataqv and Subread [30, 31]. Peak calling was performed using MACS2 with a q-value threshold of 0.05. Genes with ATAC-seq peaks overlapping transcription start sites (TSS) were identified using ChIPseeker v1.44.0 [32]. ATAC-seq peaks were overlapped with eQTLs to facilitate downstream motif analysis.

### Transcription factor binding sites analysis

Motif enrichment analysis was conducted using the Multiple EM for Motif Elicitation (MEME) Suite software (v.5.3 and Tomtom) [33]. Peaks in open chromatin regions that contain eQTLs were used as input. The gene sequences and promoter regions were retrieved using the Ensembl API package in R [34]. Tomtom was employed to compare the identified motifs against a database of known motifs, using default parameters and referencing the vertebrates and mouse databases [35]. Furthermore, the Analysis of Motif Enrichment (AME) in the MEME Suite was used to identify shared transcription factor binding motifs in the promoter sequences of *trans*-regulated genes, which may indicate common regulatory mechanisms. This approach helps pinpoint transcription factors that may coordinate the expression of multiple genes [36].

### Overlap with chromatin states using the FAANG database

The eQTLs that overlapped with ATAC-Seq peaks, and the sequences used for motif analysis, were also compared with ATAC-seq and chromatin states data from the FAANG database [11]. This comparison helped determine if the eQTLs were in active chromatin regions that could influence gene expression. Genome browser visualization of ATAC-seq peaks, eQTL loci, and FAANG chromatin state annotations were performed using the Integrative Genomics Viewer (IGV).

## Results

**Fig. 1:**
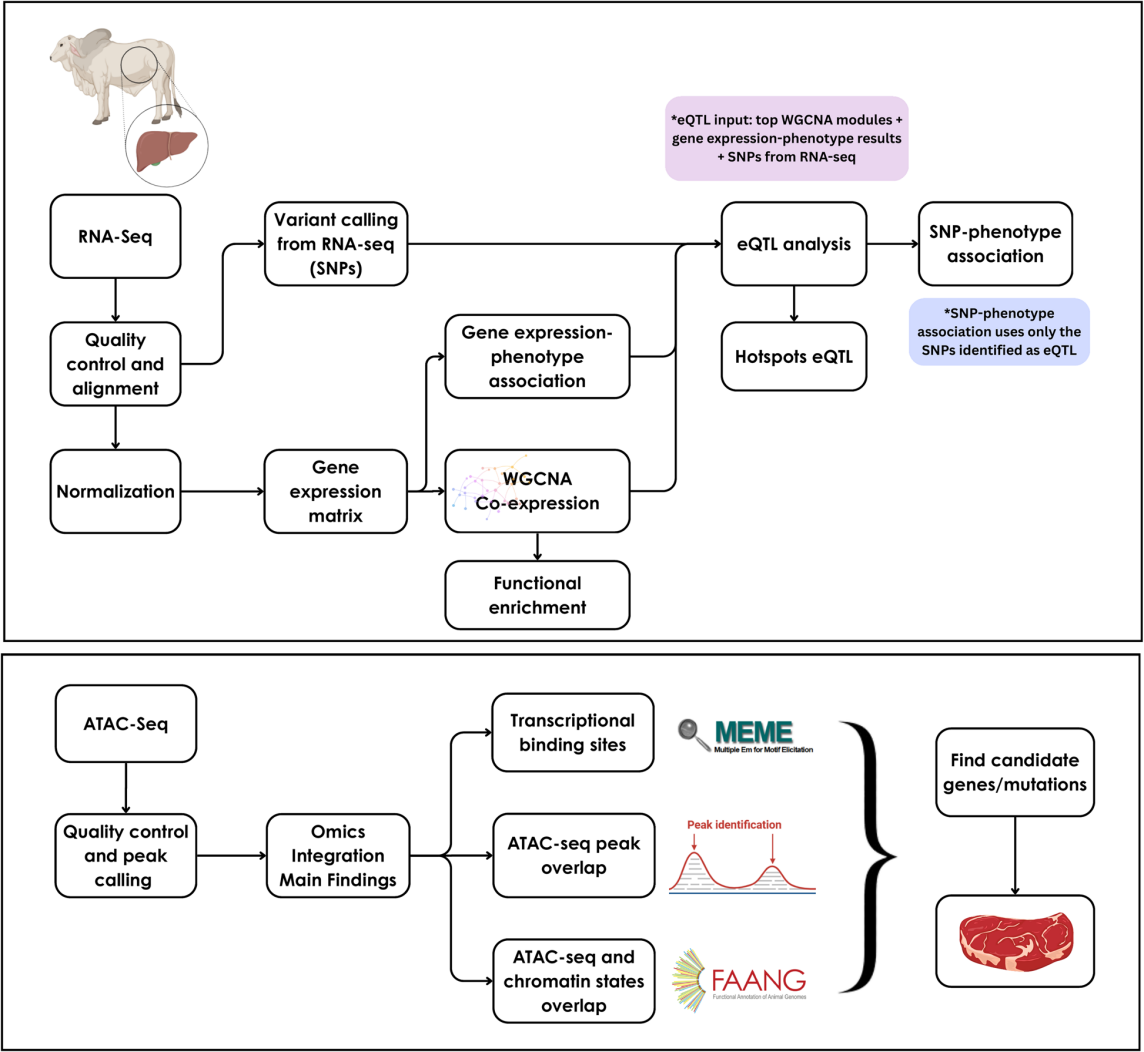
Overview of the multi-layer approach workflow used to identify regulatory mechanisms associated with meat and carcass quality traits in Nelore cattle

To investigate the regulatory mechanisms underlying meat and carcass quality traits in cattle, a multi-layer workflow was implemented, integrating gene expression, SNP discovery, eQTL analysis, phenotype association, and chromatin accessibility data (Fig. 1).

### Characterization of the transcriptomic profile of the liver

#### *RNA-seq and SNP calling*

On average, 17.2 million paired-end reads were generated per sample. After quality control, the clean reads were mapped to the bovine reference genome (UCD1.2), resulting in an average mapping rate of 87.7% (Table S1). A total of 27,570 genes were identified in the reference genome. After filtering out genes with fewer than one count in at least 50% of the samples, 17,356 genes were retained for downstream analyses (Table S1). SNP calling from the RNA-Seq data identified 146,374 biallelic SNPs, following filtering for a call rate above 95% and MAF greater than 5%.

#### *Chromatin accessibility profiling *via* ATAC-seq*

ATAC-seq data quality was assessed using standard chromatin accessibility metrics. Both liver samples showed robust data quality, with TSS enrichment scores above 4, fraction of reads in peaks (FRiP) scores exceeding 25%, high mapping quality, and no evidence of excessive PCR duplication, indicating a good signal-to-noise ratio. Detailed ATAC-seq quality control metrics are provided in Table S3.

To evaluate chromatin accessibility, ATAC-seq was performed on two liver samples, followed by peak calling using MACS2. After filtering out non-standard chromosomal regions (e.g., chrUn and chrM), an average of 149,858 peaks was identified across samples. The average peak length was approximately 250.3 bp, with the largest peaks spanning up to 3,007 bp. These regions represent accessible chromatin and were used for downstream regulatory annotation and motif analysis. Using bedtools, 82,724 peaks were shared between the two samples, indicating a substantial core of conserved accessible regions, while 53,612 peaks were unique to Liver-5.I and 83,982 peaks to Liver-10.IV samples.

To functionally characterize the genomic context of the accessible chromatin regions, we annotated the ATAC-seq peaks from both samples using CHIPseeker [32]. The annotations were based on the proximity to known gene features, including promoters, exons, introns, untranslated regions (UTRs), and intergenic regions.

In the Liver-5.I sample, the majority of peaks (64.81%) were located in distal intergenic regions, while promoter (≤ 1 kb) peaks represented 8.42%, with an additional 2.92% between 1–3 kb from the TSS. Intronic regions represented 6.39% in the first introns and 15.22% in the other introns (Fig. S2). The enrichment profile showed that 18% of peaks were located within 10 kb of the nearest TSS. However, a notable fraction of peaks extended beyond 100 kb from the closest gene, highlighting a strong distal regulatory component (Fig. S3).

The Liver-10.IV sample also showed a majority of peaks located in intergenic regions (66.72%). Promoter-associated peaks accounted for 9.96% of the total, with 7.44% located within 1 kb upstream of TSS. Intronic regions accounted for 5.97% of the first introns and 15.24% of the other introns. Only a small fraction of peaks were found in exons or UTRs (Fig. S4). The distribution of distances to TSS followed a similar pattern to the other sample within enrichment, ~ 15% of peaks were located within 10 kb of the nearest TSS, while the majority were distributed between 10–100 kb and > 100 kb from the closest gene (Fig. S5). Representative genome browser snapshots integrating ATAC-seq signal from this study with FAANG ATAC-seq and chromatin state annotations are shown in Fig. S6.

### WGCNA

**Fig. 2:**
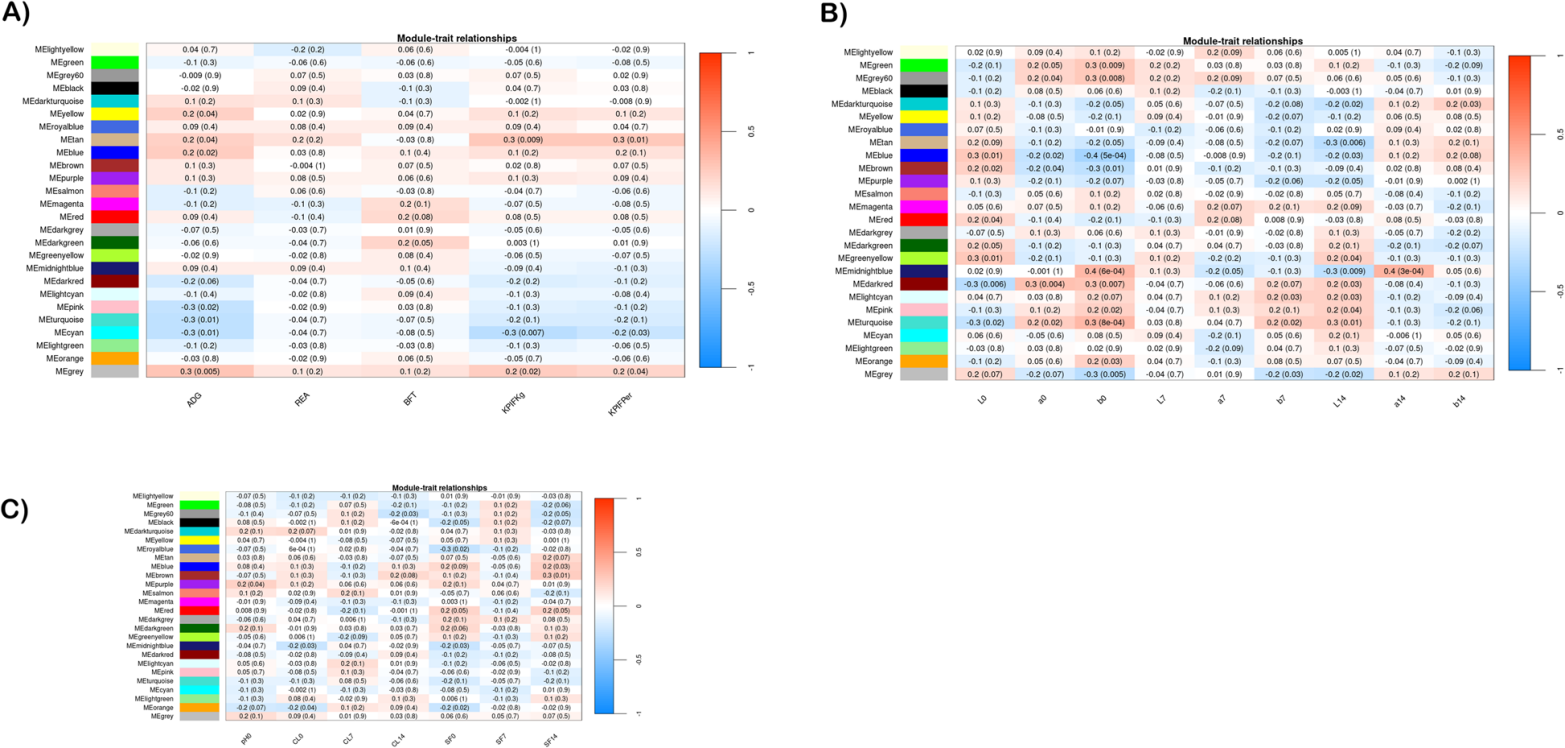
WGCNA module–trait relationships. Heatmaps showing Pearson correlations between co‑expression modules (rows) and phenotypic traits (columns), with correlation coefficients and *P*‑values indicated. Colors represent the direction and magnitude of correlations (red, positive; blue, negative). **A** Growth and carcass traits. **B** Meat color. **C** Meat quality traits

The WGCNA R package was used to identify co-expressed gene modules and their associations with the traits of interest. Based on that, we identified 25 modules, of which 10 were significantly correlated with the target traits (Table S1). Gene modules were correlated with phenotypes including color, shear force, weight gain, back fat thickness, cooking loss, kidney fat, pelvic fat, inguinal fat, and pH (*P* < 0.05; Table S1 and Figs. 2, S7, S8, and S9). Within the significant modules, hub genes were identified. Genes with a module membership higher than 0.8 were considered hubs. The only modules showing significant functional enrichment (FDR < 0.1) were blue, red, and tan.

### Energy metabolism-related pathways are over-represented in the blue module

**Fig. 3:**
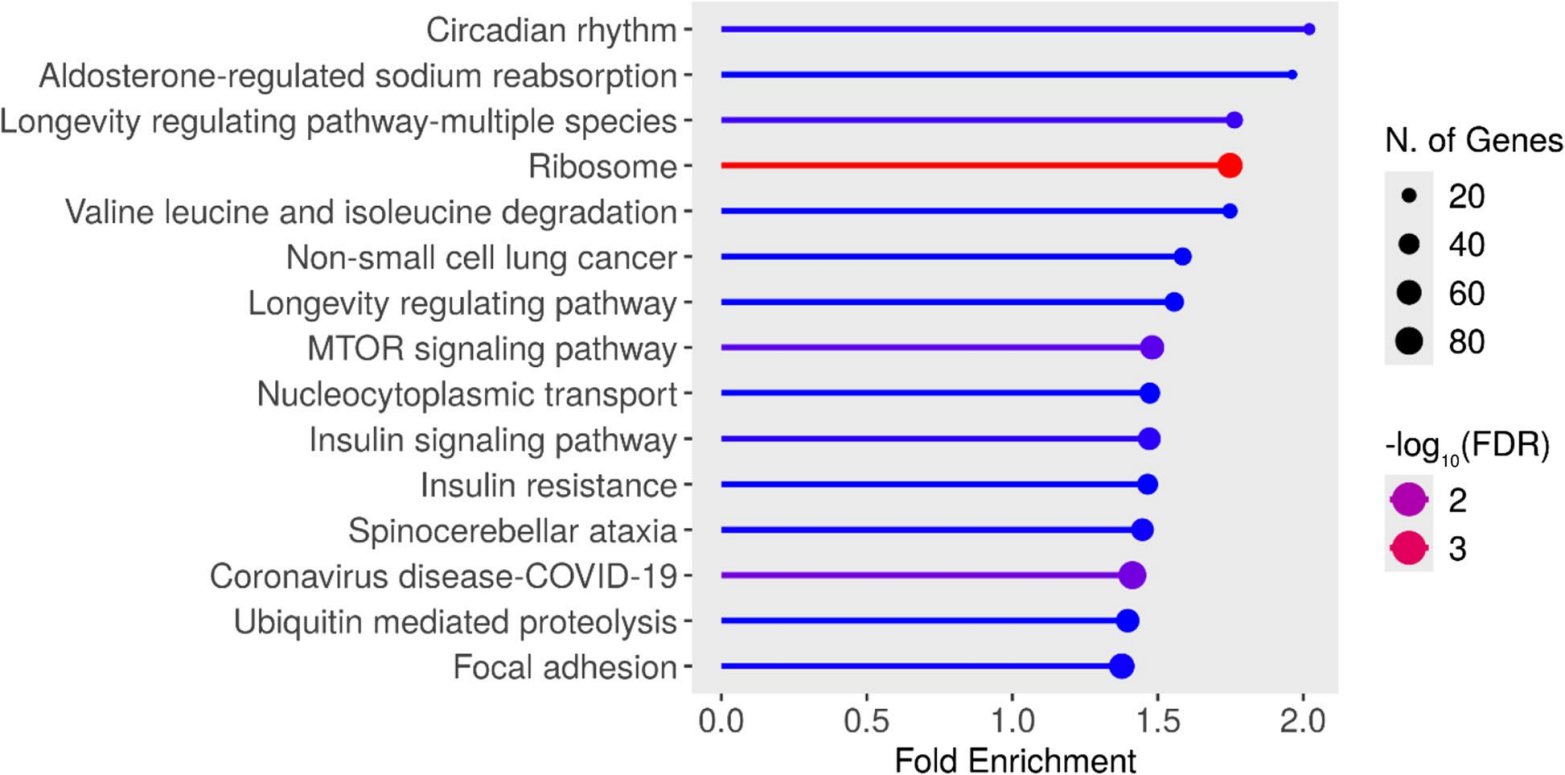
The top 20 enriched KEGG pathways from the hepatic genes of Nelore bulls are clustered within the blue module. The *x*-axis represents fold enrichment. Circle size indicates the number of genes associated with each pathway, and color represents the significance level (−log_10_ FDR), with darker colors indicating higher significance

The highest correlation was observed between the blue module and color phenotypes, with an *r* value of −0.4 for the yellowness (b*) phenotype at 24 h after slaughter (Fig. 2). Additionally, this gene module was correlated with average daily gain (ADG) and SF at 14 d after slaughter (SF14) (Fig. 2). The ShinyGO v.0.82 [21] revealed several pathways among the genes in the blue module, including ribosome (FDR = 0.0001), mTOR signaling (FDR = 0.053), insulin signaling (FDR = 0.078), circadian rhythm (FDR = 0.071), aldosterone-regulated sodium reabsorption (FDR = 0.087), and insulin resistance (FDR = 0.087), all related to energy metabolism (Fig. 3).

### Protein and lipid metabolism pathways enriched in red and tan modules

The red module was associated with SF at 24 h and 14 d after slaughter (SF0 and SF14) and with lightness of meat at 24 h (L*₀). The enrichment of the red module revealed pathways related to protein processing and metabolic activity. The most enriched pathway was protein processing in the endoplasmic reticulum (FDR = 2.42 × 10^−6^), followed by lipid and atherosclerosis (FDR = 0.0014). The tan module was associated with ADG, kidney, pelvic, and inguinal fat (KPIF), and color phenotypes. The tan module enrichment revealed pathways including the PI3K-Akt signaling pathway (FDR = 2.63 × 10^−7^), protein digestion and absorption (FDR = 2.63 × 10^−7^), and focal adhesion (FDR = 2.63 × 10^−7^) (Table S1).

### Gene expression–phenotype associations based on negative binomial modeling

To complement the WGCNA analysis, gene expression–phenotype association analysis was conducted to assess the impact of individual gene expression variation on the phenotype. We identified a significant connection between 6,311 genes and b*₀, highlighting the potential key role of liver gene expression in early meat coloration. Additionally, 948 genes were associated with L*₀, and 184 genes with lightness at 14 d (L*₁₄). Redness (a*) was associated with fewer genes: 2 at 7 d (a*₇) and 17 at 14 d (a*₁₄).

For fat deposition, we identified 15 genes related to kidney, pelvic, and inguinal fat percentage (KPIFPer) and 8 genes associated with fat in kilograms (KPIFKg). In terms of growth and carcass traits, 35 genes were associated with ADG, four with ribeye area (REA), and five with backfat thickness (BFT).

For meat tenderness, we identified 10 genes associated with SF0, two at 7 d (SF7), and three at 14 d (SF14). Cooking loss was associated with seven genes at 24 h (CL0), three at 7 d (CL7), and three at 14 d (CL14). Using the gene expression-trait approach, we identified 738 genes that were not included in the list of genes from the ten significant modules of WGCNA (Fig. S10; Table S2).

### eQTL analysis

In contrast to conventional eQTL analyses that typically evaluate all expressed genes in a tissue against variant genotypes, our approach focused on a biologically informed subset of genes. Specifically, we restricted our eQTL analysis to genes that were previously identified as relevant based on co-expression network modules and gene expression–phenotype associations. This strategy aimed to enhance the biological relevance and interpretability of the detected regulatory variants, prioritizing expression changes associated with the phenotype of interest.

Genes from the ten significantly associated modules in WGCNA, as well as genes individually associated with the traits, were selected for this analysis. The eQTL analysis included 11,578 genes and 146,374 SNPs derived from RNA-Seq data. A coding system was applied to each gene to indicate the module it originated from and the phenotype it was associated with (Table S3).

The analysis revealed 1,198 *cis-*eQTLs and 39,227 *trans-*eQTLs with FDR < 0.05 (Table S3). Among the *cis-*eQTLs, the alternative allele increased the expression of 236 genes, while reducing the expression of 960 genes. In the *trans-*eQTLs, the alternative allele increased the expression of 2,304 genes while decreasing the expression of 36,923 genes.

The functional classification using VEP revealed that 25% of the variants were annotated as synonymous, followed by missense (14.6%), 5′ UTR (8.3%), and 3′ UTR (6.3%). A considerable portion was found in intronic regions (18.8%), while upstream (8.3%) and downstream gene variants (8.3%) were also represented. A smaller fraction (0.1%) corresponded to intergenic variants.

### Hotspot eQTLs

To pinpoint eQTLs affecting the expression levels of multiple genes, a permutation test was performed (Table 1; Table S3). The hotspot eQTL rs449155362 on chromosome 25 regulates 848 genes (*P* = 0.00014). The eQTLs rs525034686, rs451052057, and rs515950699 are in the same LD block (Table S3). Notably, all 5 top eQTL hotspots were located on chromosome 25 (Table 1), suggesting a major regulatory hub.

**Table 1:** The top five eQTL hotspots identified in the present study

rsID	Position	Chr	*Cis*	*Trans*	Total of genes	*P*
rs525034686	26,127,340	25	19	702	721	0.00044
rs451052057	26,127,361	25	19	702	721	0.00044
rs515950699	26,127,500	25	19	494	513	0.00074
rs110051702	27,995,286	25	3	513	516	0.00059
rs449155362	33,826,762	25	8	840	848	0.00014

Each row represents an SNP that acts as an expression quantitative trait locus (eQTL) hotspot. Columns indicate: rsID (SNP identifier), Position (genomic position in base pairs), Chr (chromosome), *Cis* (number of genes with *cis* associations to the SNP), *Trans* (number of genes with *trans* associations), Total of genes (sum of *Cis* and *trans* associations), and *P* (significance level of the hotspot)

### SNP-trait association

SNP-phenotype associations were evaluated for variants previously identified as eQTLs regulating genes associated with meat and carcass quality traits. Overall, this analysis identified 54 SNPs significantly associated with REA, BFT, meat yellowness (b*₀, b*₁₄), and pH24 (FDR < 0.05) (Table S3). Most associations were related to BFT, whereas the WGCNA and gene expression–phenotype analyses identified only a single module correlated with this trait.

**Fig. 4:**
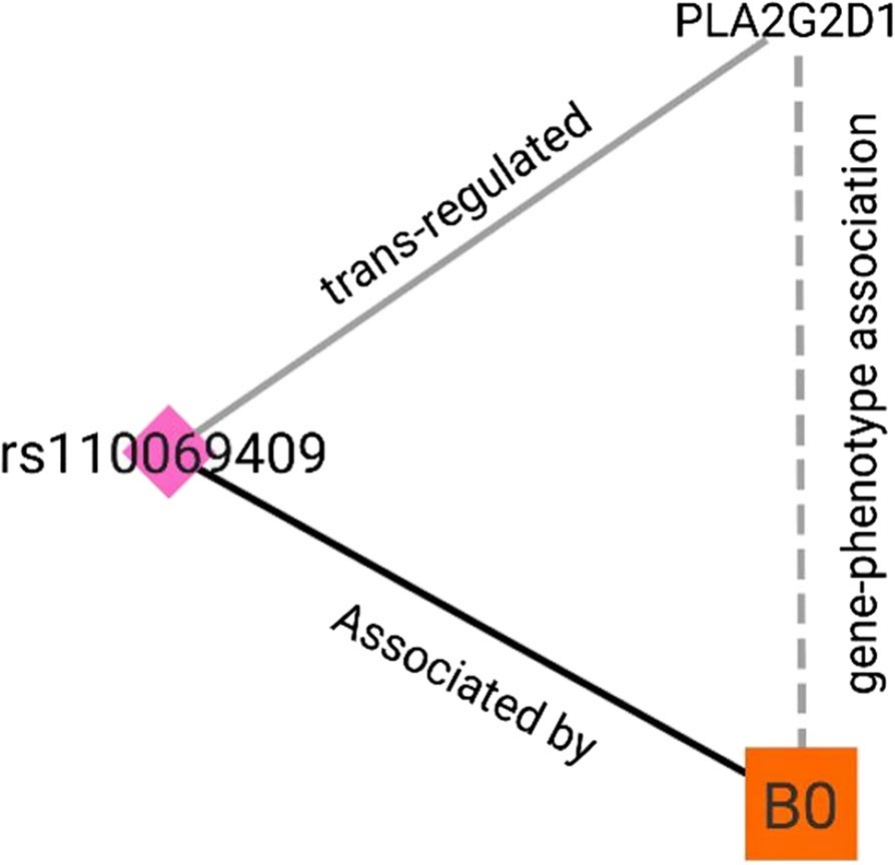
Multi-layer evidence of genetic regulation for the b*₀ phenotype (yellowness at 24 h postmortem), labeled as “B0” in the figure. The rs110069409 (eQTL) regulates *PLA2G2D1*; this gene is associated with the Deseq2 package (gene expression-phenotype) with b*₀, and the rs110069409 is also associated with b*₀ by the SNP-phenotype analysis

Among the gene expression–phenotype and SNP–phenotype approaches, only one variant on chromosome 3 showed concordant association with the same phenotype at both levels. Specifically, rs110069409, an intronic variant regulating *PLA2G2D1* in *trans*, was significantly associated with b*₀ (FDR = 0.02825), consistent with the association observed for *PLA2G2D1* expression (Fig. 4).

On chromosome 21, three missense SNPs (rs1116460545, rs460498788, and rs479604354) were significantly associated with BFT (FDR < 0.05). Although these variants were located within the *IFI27I* and *IFI27L2* genes, their regulatory effects were observed on the target genes *BEND6* and *CPNE5*, respectively. These target genes were assigned to the cyan and turquoise WGCNA modules, which were associated with traits including ADG, meat color, and KPIF. In addition, the rs714368767 missense variant associated with BFT regulates *USP13*, a gene in the blue module, which is associated with ADG, b*₀, and SF14.

### Integration analysis of transcription factor binding sites, ATAC-seq, and chromatin states

To further explore the regulatory architecture underlying phenotypic variation, we integrated the results from eQTL hotspots, SNP-trait associations, ATAC-seq, and chromatin state annotations. In the following sections, we will describe the main results.

#### PLA2G2D1 and its negative association with the b*₀ color phenotype

For the b*₀ phenotype, the eQTL rs1100069409 emerged as a key integrative element between the gene-trait and SNP-trait approach. This variant regulates the gene *PLA2G2D1* in *trans*. Individuals carrying the homozygous alternative genotype (1/1) exhibit significantly higher *PLA2G2D1* expression than the reference (β = 1.09, *P* = 0.0047). Notably, *PLA2G2D1* expression is negatively associated with b*₀, as determined by a negative binomial regression. rs1100069409 is the only case where both layers of evidence converged. Furthermore, this eQTL is located within an ATAC-seq peak and in a chromatin state region associated with an active TSS.

#### MLXIPL as a regulatory hub linking growth and meat color

The eQTL hotspot rs449155362, located on chromosome 25, regulated 848 genes, including the *MLXIPL* transcription factor (TF) in *cis*. *MLXIPL* is a hub gene in the blue module, which shows a negative correlation with b*₀ and L*₁₄ and a positive correlation with ADG, SF14, and L*₀. A chromatin peak does not show an overlap, but the chromatin state of CTCF binding and active TSS from the FAANG database overlapped with this eQTL. To further investigate this finding, we conducted a motif enrichment analysis of the promoter regions of genes regulated by rs449155362 to identify transcription factors that may be involved in their expression. The promoter sequences for 800 of these genes were successfully obtained using the Ensembl API [34].

After that, we used the Analysis of Motif Enrichment (AME) from the MEME Suite software [33] to identify the specific transcription factors that bind to these gene promoters. This analysis revealed binding sites for several transcription factors, including *MLXIPL* (Table S3). The analysis indicated that *MLXIPL* binds to the promoter regions of the *trans*-regulated genes. The binding site for *MLXIPL* had a *P* = 2.34 × 10^−5^ with an *P*_adj_ = 2.88 × 10^−2^. The observed binding score was 1.08, indicating a moderate level of enrichment at the binding site (Table S3).

## Discussion

In the context of modern livestock genetics, integrating a multi-layered approach is essential for unraveling the regulatory mechanisms underlying economically important traits. This study contributes to that effort by combining transcriptomic profiling, variant mapping, and chromatin accessibility data to characterize the liver's role in meat and carcass quality traits in cattle.

Among the evaluated phenotypes, meat color showed the strongest associations with gene expression, suggesting that liver transcriptional activity was closely related to postmortem color development. In contrast, SNP–phenotype associations based on expressed variants from RNA-seq were more enriched for backfat thickness, reflecting genetic components linked to lipid metabolism.

The present work found ten significant co-expressed gene modules associated with the phenotypes. Within those modules, the blue module showed the highest correlation with the color b*₀ phenotype. The blue module is correlated with ADG (0.2), L*₀ (0.3), SF14 (0.2), a*₀ (−0.2), b*₀ (−0.4), and L*₁₄ (−0.3). The functional enrichment of the blue module revealed pathways like circadian rhythm, aldosterone-regulated sodium reabsorption, mTOR, ubiquitin-mediated proteolysis, insulin, and insulin resistance.

The enrichment of circadian rhythm and aldosterone-regulated sodium reabsorption pathways in the blue module suggests that systemic physiological regulation may influence hepatic transcriptional activity linked to meat quality traits. Several genes enriched in the circadian pathway, such as *CREB1*,* CRY1*,* PRKAA2*,* NPAS2*, and *RORC*, are known to regulate metabolic rhythms and energy homeostasis in peripheral tissues, including the liver and muscle [37–40]. For instance, *CREB1* acts as a central transcription factor that modulates lipid metabolism and insulin sensitivity, both of which can be key determinants of postmortem meat color and oxidative stability [38].

Complementing these findings, the identification of aldosterone-regulated sodium reabsorption as an enriched pathway in the hepatic transcriptome of Nelore cattle suggests that hormonal and ion-transport mechanisms may contribute to the regulation of meat quality traits. Genes such as *IGF1, PIK3CA, MAPK1, INSR,* and *NR3C2*, which are part of this pathway, are central to metabolic signaling and energy regulation. Notably, *SGK1* and *NR3C2* are key mediators of aldosterone action, promoting sodium retention and cellular hydration [41]. These processes can be essential for maintaining water-holding capacity, which may impact the meat color and tenderness.

Further supporting the metabolic relevance of the blue module, several genes are enriched in the mTOR pathway. This pathway can be indirectly stimulated by *IGF-1* and *P70S6K*, thereby initiating protein synthesis [42, 43]. Some of the genes enriched in the mTOR pathway were *IGF1*,* EIF4B*,* PIK3CA*,* LAMTOR4*,* PRKAA2*,* PRKACB*,* GRB2*,* INSR*, and *SLC38A9*. The modulation of the mTOR pathway by insulin, as well as by other hormones and nutrients, contributes to enhanced protein synthesis [44, 45]. The blue module also revealed pathways such as insulin signaling and insulin resistance, and some of the genes involved included *INSR, PIK3CA, PRKACB, FOXO1, PHKG2, MAPK1, PRKAA2, MLXIPL, CREB1, PPARA, CREB3L4*, and *MAPK9.*

The connections among metabolic signals involved in appetite control (such as propionate), glucose metabolism, and insulin secretion are complex and warrant consideration when discussing the regulation of dry matter intake. Propionate's function highlights the significance of these elements in signaling fullness in ruminants [45–47], while also acting as the main precursor for gluconeogenesis [45, 48], stimulating insulin release [45, 49], and possibly contributing to insulin resistance [45, 47].

Foote et al. [45] showed that steers with higher ADG exhibited a delayed peak insulin response and an elevated glucose nadir following an intravenous glucose tolerance test (IVGTT), suggesting impaired insulin sensitivity. When considering our findings, the positive correlation between ADG and the blue module, which was enriched for insulin and insulin resistance, reinforces the hypothesis that metabolic signals influencing insulin dynamics may also play a role in regulating growth.

Shifting focus from growth-related traits, gene expression–phenotype associations also revealed important links to meat color parameters. Meat color, frequently regarded as a sign of meat freshness and quality, is a key factor influencing consumer buying decisions [50, 51]. Typically, the color of meat is assessed objectively using a spectrophotometer, and its values are articulated in terms such as lightness (L*), redness (a*), and yellowness (b*). These characteristics have gained significance in genetic selection as metrics for meat quality, considering that consumers tend to pay a premium for meat products exhibiting superior intrinsic quality [50].

According to Virtuoso et al. [52], the genes *GNPDA2*,* CALCRL*,* CALM1*,* KIT*,* PRKN*, and *PRKAA2* are the most promising candidates associated with L*. *PRKAA2* is in the blue module and underlying the mTOR and insulin resistance pathway. This gene plays a crucial role in the metabolism of reactive oxygen species, lipids, and carbohydrates, as well as in glycolysis. The study suggests that the gene also plays a significant role in determining the lightness of meat (L*) [52]. Genes associated with meat yellowness (b*) in the gene expression–phenotype association—including *HSF4*,* CTCF*,* AFF3*,* NFAT5*, and *THAP11*—are located within or near genomic regions previously reported in a study of multi-trait GWAS for meat color traits in Nelore cattle [53].

These findings suggest that hepatic gene expression may indirectly influence muscle phenotypes through systemic metabolic mechanisms. The liver plays a central role in regulating lipid and carbohydrate metabolism, which can influence muscle metabolism and, in turn, affect meat quality traits.

### *PLA2G2D1* is negatively associated with the b*₀ trait

We identified an intronic variant, rs110069409, that regulates *PLA2G2D1* expression in *trans* and is significantly associated with the meat color trait b*₀. Notably, *PLA2G2D1* expression levels were also correlated with b*₀, supporting its involvement in the molecular mechanisms underlying variation in meat color.

Members of the phospholipase A2 (PLA2) gene family are low-molecular-mass esterases that hydrolyze glycerophospholipids, generating free fatty acids and lysophospholipids [53]. Previous studies in pigs [54] and chickens [55] have shown that increased PLA2 activity under cellular stress, such as elevated intracellular calcium levels, can promote lipid oxidation and contribute to meat quality defects, including altered color and reduced oxidative stability. These observations suggest that variation in PLA2-related pathways may influence meat color traits through lipid oxidative processes.

The SNP rs110069409, which regulates *PLA2G2D1* in *trans*, was found within an ATAC-seq peak and overlapped an active TSS (E2) chromatin state. Active TSS regions are typically associated with accessible chromatin and active transcription initiation, indicating that this variant lies within a regulatory locus with potential functional activity [9, 11]. In the context of a *trans-*eQTL, this suggests an indirect regulatory mechanism whereby the SNP may affect the expression of a nearby gene or regulatory factor that subsequently modulates *PLA2G2D1* expression through downstream regulatory networks.

Taken together, these results indicate that variation in *PLA2G2D1* expression may contribute to differences in meat color, potentially through systemic effects related to lipid metabolism and oxidative balance. The opposing associations observed among rs110069409, *PLA2G2D1* expression, and the b*₀ phenotype may reflect compensatory regulatory mechanisms or complex network-level interactions rather than direct *cis*-regulatory effects.

### *MLXIPL* as a regulatory hub linking growth and meat color

The eQTL rs449155362 regulated the expression of 848 genes, with eight in *cis* (*EIF4H*, *BUD23, RASA4B, NSUN5, TMEM120A, LAT2, LRWD1*, and *MLXIPL*). Among them, the TF *MLXIPL*, also known as *ChREBP*, is involved with glucose metabolism. *MLXIPL* works with its partner *Mlx* to mediate a significant portion of the transcriptional response triggered by glucose [56]. They accomplish this by binding to the promoters of target genes that contain carbohydrate response elements (ChoREs) [57]. Under high-glucose conditions, *Mlx* complexes accumulate in the nucleus. There, they bind to the promoters of target genes, regulating gene expression in response to metabolic signals. This complex is recognized as one of the primary regulators of gene expression induced by glucose [57, 58].

*MLXIPL* is central in glucose and lipid metabolism in various tissues, including muscle [59]. In particular, muscle color, which can be influenced by glycolytic rate and glycogen storage, might be indirectly impacted by the metabolic functions of these genes in the liver. Therefore, the observed expression patterns in liver tissue could relate to muscle characteristics, including color, which is relevant to meat quality [60].

The *MLXIPL* was identified as a hub gene in the blue WGCNA module, showing a positive correlation with ADG and L*₀, while exhibiting negative correlations with b*₀ and a*₇. Additionally, *MLXIPL* is involved with the insulin resistance KEGG pathway, underscoring its significance in overall glucose and insulin dynamics.

The influence of rs449155362 on many genes, likely through the regulation of *MLXIPL*, highlights its potential impact on metabolic pathways and glucose-responsive gene expression. Although rs449155362 is located distally, it may act through long-range regulatory interactions. Even though rs449155362 was not found in an accessible chromatin region, which may also be due to the limited sample size used in this study, its position in a CTCF/Active TSS state suggests it could play a role in long-range regulatory interactions. CTCF has been implicated in the formation of chromatin loops in various systems, bringing distal regulatory elements into proximity with promoter sequences [60, 61].

While combining SNP-phenotype and gene expression–phenotype associations yielded valuable insights, this study has limitations that warrant acknowledgment. The SNPs used for association analysis were derived from RNA-seq data, which, while informative, represent only the transcribed portion of the genome. This restricts variant discoveries to expressed genes, potentially missing regulatory variants in non-coding regions. Additionally, using only one tissue narrows the scope of regulatory inference, potentially missing important interactions in muscle or fat tissue, both of which are critical for carcass and meat quality traits. Genotype imputation was not performed, which limits SNP density and may have missed regulatory variants outside transcribed regions. The focus of this study was on prioritizing functionally supported regulatory candidates, not on comprehensive variant detection, and future work incorporating whole-genome genotypes would improve variant coverage and enable more rigorous colocalization analyses.

Although ATAC-seq data added a functional dimension by capturing chromatin accessibility, it was collected from only two animals, limiting statistical power and generalizability. Additionally, factors like sample size and population structure might have affected the detection and interpretation of associations. Future studies should broaden the range of tissues, increase sample sizes for functional tests, and use integrated multi-omics strategies to better understand the molecular basis of complex traits in cattle.

## Conclusion

We integrated gene expression–trait associations, eQTL mapping, co-expression networks, and chromatin features to provide a multilayered view of the regulatory architecture underlying beef quality. From our analysis, *PLA2G2D1* emerged as a candidate regulator of meat color, especially b*₀, supported by convergent evidence from eQTL and chromatin accessibility findings.

Additionally, *MLXIPL* was identified as a hub gene in the blue module, linking growth, carcass, and meat-quality-related traits, including ADG and SF14, to meat color traits such as b*₀, L*₀, L*₁₄, and a*₀. Functional enrichment analyses suggest that this module is associated with metabolic regulation pathways, including insulin resistance and mTOR signaling. These findings highlight the complexity of genetic regulation, in which loci may contribute to both growth efficiency and meat quality through interconnected regulatory networks. Although additional validation in muscle tissue and functional assays will be required to confirm specific molecular mechanisms, our results underscore the value of multi-omics integration for prioritizing candidate loci and providing a framework for future genetic selection strategies aimed at improving carcass and meat quality.

## Supplementary Information

Additional file 1: Fig. S1. Graphic representation of the first two principal components of the population stratification test. Fig. S2. Genomic annotation of ATAC-seq peaks in liver tissue (sample Liver 5-I) from Nelore cattle. Fig. S3. Distribution of transcription factor binding sites relative to transcription start sites (TSS) in liver tissue (sample Liver 5-I) from Nelore cattle. Fig. S4. Genomic annotation of ATAC-seq peaks in liver tissue (sample Liver 10-IV) from Nelore cattle. Fig. S5. Distribution of transcription factor binding sites relative to transcription start sites (TSS) in liver tissue (Liver 10-IV sample) from Nelore cattle. Fig. S6. Chromatin landscape of selected eQTL SNPs in bovine liver. Fig. S7. Sample dendrogram and heatmap of carcass quality traits. Fig. S8. Sample dendrogram and Heatmap of meat quality traits. Fig. S9. Sample dendrogram and heatmap of meat color traits. Fig. S10. Venn Diagram of the ten significant modules of WGCNA with the gene expression-phenotype analysis.

Additional file 2: Table S1-1. Summary of RNA sequencing and mapping statistics. Table S1-2. Minimum, maximum, average, and standard deviation for meat and carcass quality phenotypes in Nelore cattle. Table S1-3. Phenotypes corrected (residuals) for Contemporary groups (Slaughter + days in confinement), age, PC1 and PC2. Table S1-4. Statistically significant modules of the weighted correlation network analysis (WGCNA) with *P* < 0.5 and correlated phenotypes. Table S1-5. Genes for each module of the WGCNA considering only the ten significant ones. Table S1-6. Hub genes for the significant WGCNA modules.

Additional file 3: Table S2-1. Gene expression–phenotype asociation for average daily gain (ADG). Table S2-2. Gene expression–phenotype association for ribeye area (REA). Table S2-3. Gene expression–phenotype association for backfat thickness (BFT). Table S2-4. Gene expression–phenotype association for kidney, pelvic, and inguinal fat in Kg (KPIFKg). Table S2-5. Gene expression–phenotype association for kidney, pelvic, and inguinal fat in percentual (KPIFPer). Table S2-6. Gene expression–phenotype association for cooking loss at 24 hours post mortem (CL0). Table S2-7. Gene expression–phenotype association for cooking loss at 7 days post mortem (CL7). Table S2-8. Gene expression–phenotype association for cooking loss at 14 days post mortem (CL14). Table S2-9. Gene expression–phenotype association for shear force 24 hours post mortem (SF0). Table S2-10. Gene expression–phenotype association for shear force 7 days post mortem (SF7). Table S2-11. Gene expression–phenotype association for shear force 14 days post mortem (SF14). Table S2-12. Gene expression–phenotype association for lightness at 24 hours post mortem (L0). Table S2-13. Gene expression–phenotype association for lightness at 7 days post mortem (L7). Table S2-14. Gene expression–phenotype association for lightness at 14 days post mortem (L14). Table S2-15. Gene expression–phenotype association for redness 7 days post mortem (a7). Table S2-16. Gene expression–phenotype association for redness 14 days post mortem (a14). Table S2-17. Gene expression–phenotype association for yellowness 24 hours post mortem (b0). Table S2-18. Gene expression–phenotype association for yellowness 14 days post mortem (b14)

 Additional file 4: Table S3-1. *Cis*-eQTL results. Table S3-2. *Trans*-eQTLs results. Table S3-3. eQTL hotspots (permutation analysis). Table S3-4. SNP x Phenotype association results. Table S3-5. Ame enrichment results of the promoter region of the *trans* regulated genes of the rs449155362 eQTL. Table S3-6. MLXIPL target genes identified by AME enrichment for rs449155362. Table S3-7. ATAC-seq Quality Control Metrics. Table S3-8. ATAC-seq results for Liver-5.I sample. Table S3-9. ATAC-seq results for Liver-10.IV sample. Table S3-10. LD results using PLINK. Table S3-11. Overlap of the eQTLs in *cis* with ATAC-seq peaks. Table S3-12. Overlap of the eQTLs in *trans* with ATAC-seq peaks. Table S3-13. Overlap of the *cis*-eQTLs with chromatin states. Table S3-14. Overlap of the *trans*-eQTLs with chromatin states. Table S3-15. Integration of main findings.

## Data Availability

The sequencing data generated in this study have been deposited in the European Nucleotide Archive (ENA) under the project accession number PRJEB102652. The data are currently restricted to private access and will be made publicly available upon acceptance of the manuscript.

## Abbreviations

ADG: Average daily gain
AME: Analysis of Motif Enrichment
ATAC-seq: Assay for transposase-accessible chromatin using sequencing
BFT: Backfat thickness
CCW: Cold carcass weight
CG: Contemporary group
CL: Cooking loss
CPM: Counts per million
CW: Carcass weight
DP: Dressing percentage
eQTL: Expression quantitative trait locus
FAANG: Functional Annotation of Animal Genomes
FDR: False discovery rate
GLM: Generalized linear model
GVCF: Genomic Variant Call Format
IGV: Integrative Genomics Viewer
KPIF: Kidney, pelvic, and inguinal fat
LD: Linkage disequilibrium
MAF: Minor allele frequency
MEME: Multiple EM for Motif Elicitation
QTL: Quantitative trait locus
REA: Ribeye area
RIN: RNA integrity number
RNA-seq: RNA sequencing
SF: Shear force
SNP: Single nucleotide polymorphism
TSS: Transcription start site
UTR: Untranslated region
VEP: Variant effect predictor
WGCNA: Weighted gene co-expression network analysis
