# Supplementary material for: Transcriptomic and regulatory landscape of liver tissue associated with meat and carcass quality traits in cattle
Source: J Anim Sci Biotechnol. 2026 Aug 1;17:150. doi: 10.1186/s40104-026-01465-0 (PMC13428424; doi:10.1186/s40104-026-01465-0)
Supplement: Supplementary file 1 — Additional file 1: Fig. S1. Graphic representation of the first two principal components of the population stratification test. Fig. S2. Genomic annotation of ATAC-seq peaks in liver tissue (sample Liver 5-I) from Nelore cattle. Fig. S3. Distribution of transcription factor binding sites relative to transcription start sites (TSS) in liver tissue (sample Liver 5-I) from Nelore cattle. Fig. S4. Genomic annotation of ATAC-seq peaks in liver tissue (sample Liver 10-IV) from Nelore cattle. Fig. S5. Distribution of transcription factor binding sites relative to transcription start sites (TSS) in liver tissue (Liver 10-IV sample) from Nelore cattle. Fig. S6. Chromatin landscape of selected eQTL SNPs in bovine liver. Fig. S7. Sample dendrogram and heatmap of carcass quality traits. Fig. S8. Sample dendrogram and Heatmap of meat quality traits. Fig. S9. Sample dendrogram and heatmap of meat color traits. Fig. S10. Venn Diagram of the ten significant modules of WGCNA with the gene expression-phenotype analysis. [file 40104_2026_1465_MOESM1_ESM.docx]

**Supplementary figures**

**Transcriptomic and Regulatory Landscape of Liver Tissue Associated with Meat and Carcass Quality Traits in Cattle**

Thais Ribeiro da Silva¹; Juliana Afonso^2^; Bárbara Silva-Vignato¹; Ingrid Soares Garcia¹; Beatriz Delcarme Lima¹; Heidge Fukumasu^3^; Saulo Luz Silva^3^; Juliana Petrini^4^; Carolina Purcell Goes^1^; Bruna Petry^5^; Wellison J. S. Diniz^6^; Aline Silva Mello Cesar¹; Gerson Barreto Mourão¹; James E. Koltes^5^; Luciana Correia de Almeida Regitano^2^; Luiz Lehmann Coutinho¹


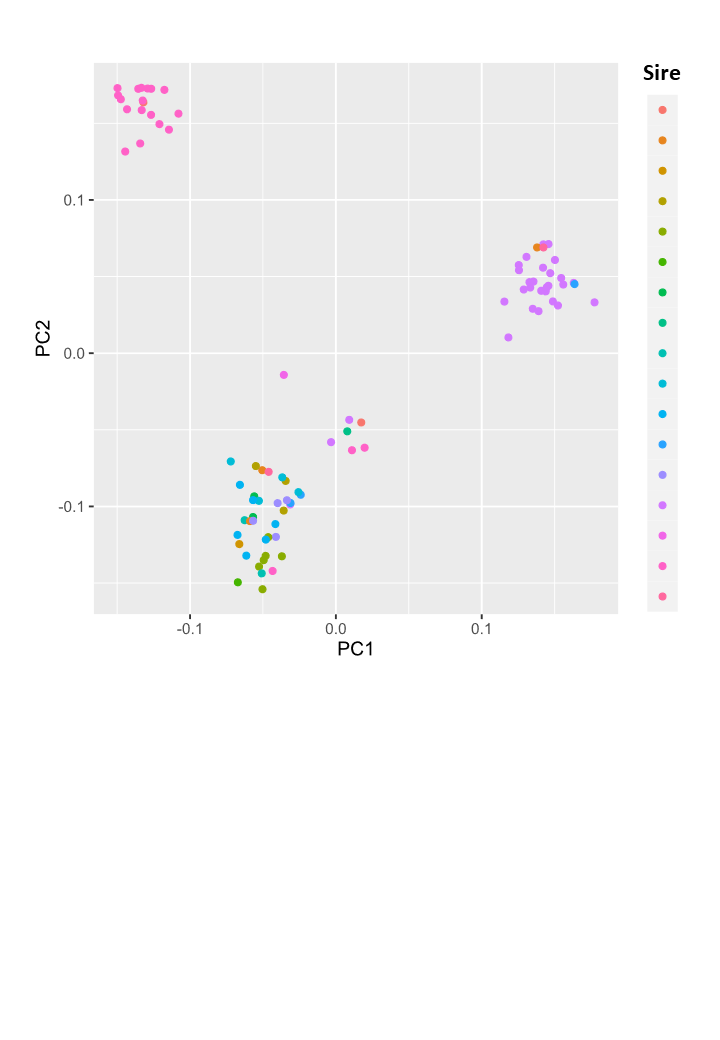


**Fig. S1- Graphic representation of the first two principal components of the population stratification test.**

The scatter plot shows the distribution of samples based on the first two principal components (PC1 and PC2) calculated from SNP genotypes derived from RNA‑seq data. Each point represents an individual animal, and colors indicate different sires, which were used for visualization purposes only. PCA was performed to assess population structure and its potential influence on downstream analyses. Clustering patterns reflect genetic relatedness among animals sharing the same sire.


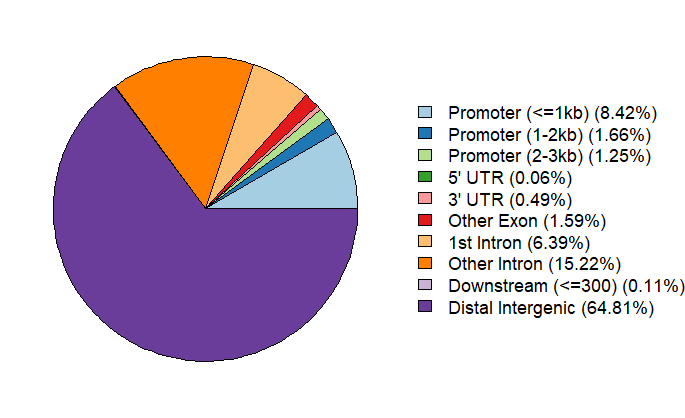


**Figure S2 – Genomic annotation of ATAC-seq peaks in liver tissue (sample Liver 5-I) from Nelore cattle.**

The pie chart shows the distribution of ATAC-seq peaks across genomic regions annotated using the ChIPseeker package. Categories include promoters at different distances from the transcription start site (≤1 kb, 1–2 kb, 2–3 kb), untranslated regions (5′ UTR, 3′ UTR), exons, introns, downstream regions (≤300 bp), and distal intergenic regions. Percentages indicate the proportion of peaks in each category, with distal intergenic regions accounting for the majority (64.81%), followed by promoters (8.42%) and intronic regions (15.22%).


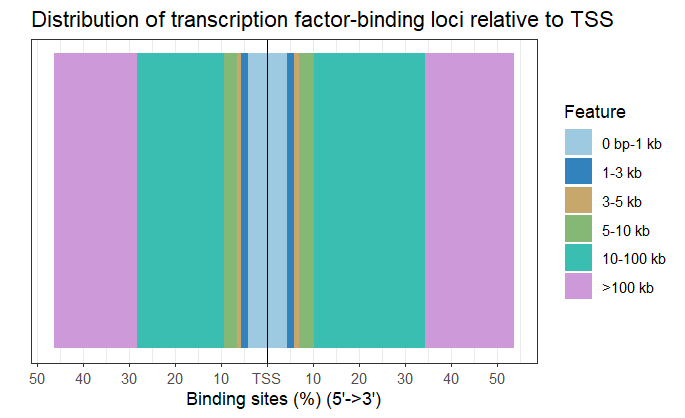


**Figure S3 – Distribution of transcription factor binding sites relative to transcription start sites (TSS) in liver tissue (sample Liver 5-I) from Nelore cattle.**

The bar plot shows the percentage distribution of transcription factor binding sites across genomic regions varying distances from the transcription start site (TSS). Categories include 0 bp–1 kb, 1–3 kb, 3–5 kb, 5–10 kb, 10–100 kb, and >100 kb from the TSS. Binding site positions were determined from ATAC-seq data and annotated to assess the proximity of regulatory elements to gene promoters.


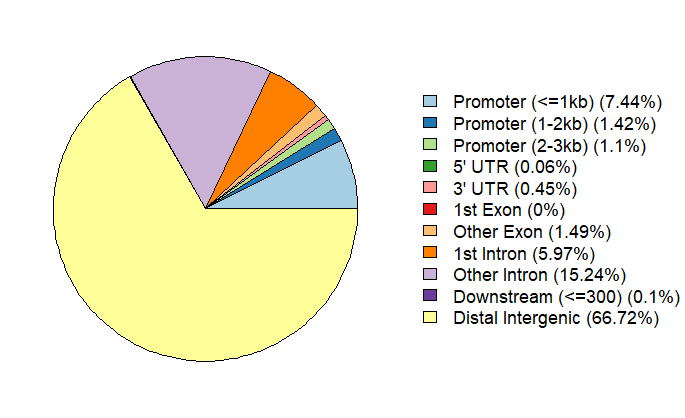


**Figure S4- Genomic annotation of ATAC-seq peaks in liver tissue (sample Liver 10-IV) from Nelore cattle.**

The pie chart shows the distribution of ATAC-seq peaks across genomic regions annotated using the ChIPseeker package. Categories include promoters at different distances from the transcription start site (≤1 kb, 1–2 kb, 2–3 kb), untranslated regions (5′ UTR, 3′ UTR), exons, introns, downstream regions (≤300 bp), and distal intergenic regions. Percentages indicate the proportion of peaks in each category, with distal intergenic regions accounting for the majority (66.72%), followed by promoters (7.44%) and intronic regions (15.24%).


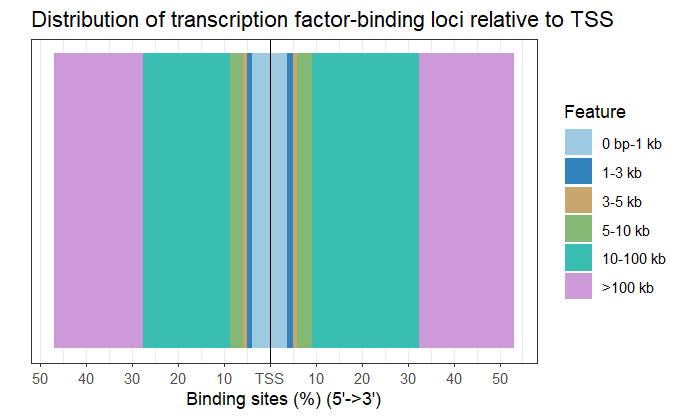


**Figure S5- Distribution of transcription factor binding sites relative to transcription start sites (TSS) in liver tissue (Liver 10-IV sample) from Nelore cattle.**

The pie chart shows the distribution of ATAC-seq peaks across genomic regions annotated using the ChIPseeker package. Categories include promoters at different distances from the transcription start site (≤1 kb, 1–2 kb, 2–3 kb), untranslated regions (5′ UTR, 3′ UTR), exons, introns, downstream regions (≤300 bp), and distal intergenic regions. Percentages indicate the proportion of peaks in each category, with distal intergenic regions accounting for the majority (66.72%), followed by promoters (7.44%) and intronic regions (15.24%).


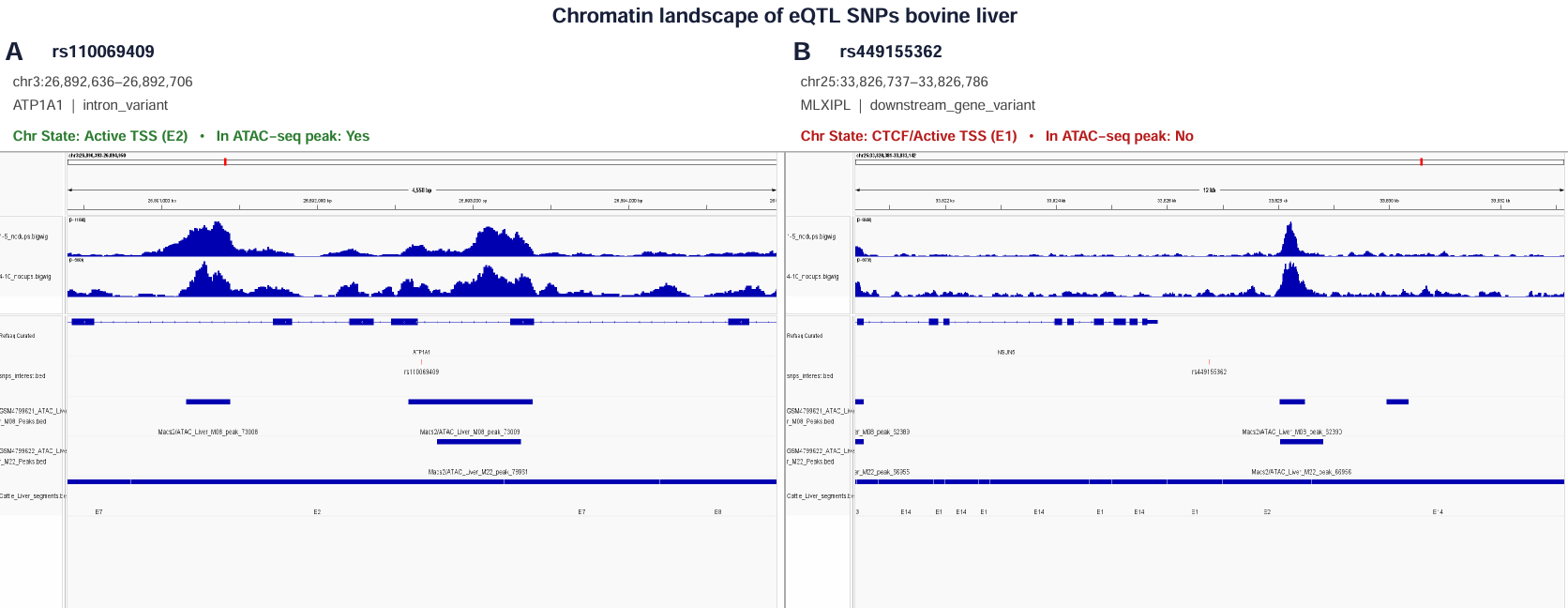


**Figure S6. Chromatin landscape of selected eQTL SNPs in bovine liver**

Genome browser views illustrate the chromatin context of two eQTL SNPs identified in Nelore cattle liver. **(A)** rs110069409 (chr3:26,892,671), a *trans‑eQTL* intronic variant regulating *ATP1A1*, located within an Active Transcription Start Site chromatin state (Active TSS; E2) and overlapping an ATAC‑seq peak. The ATAC‑seq signal is supported by both the present study (combined peaks from liver samples 5‑I and 10‑IV) and independent FAANG liver ATAC‑seq datasets (MACS2 peaks from samples M08 and M22), indicating convergent chromatin accessibility evidence across datasets. **(B)** rs449155362 (chr25:33,826,762), a *cis‑eQTL* downstream variant regulating *MLXIPL*, located within a CTCF/Active TSS chromatin state (E1) based on FAANG annotations, but not overlapping any significant ATAC‑seq peak in either the present dataset or FAANG liver ATAC‑seq data. Tracks shown (from top to bottom) correspond to ATAC‑seq signal (bigWig), RefSeq curated gene annotation, SNP position, ATAC‑seq peak calls, and chromatin state segmentation derived from FAANG liver data.


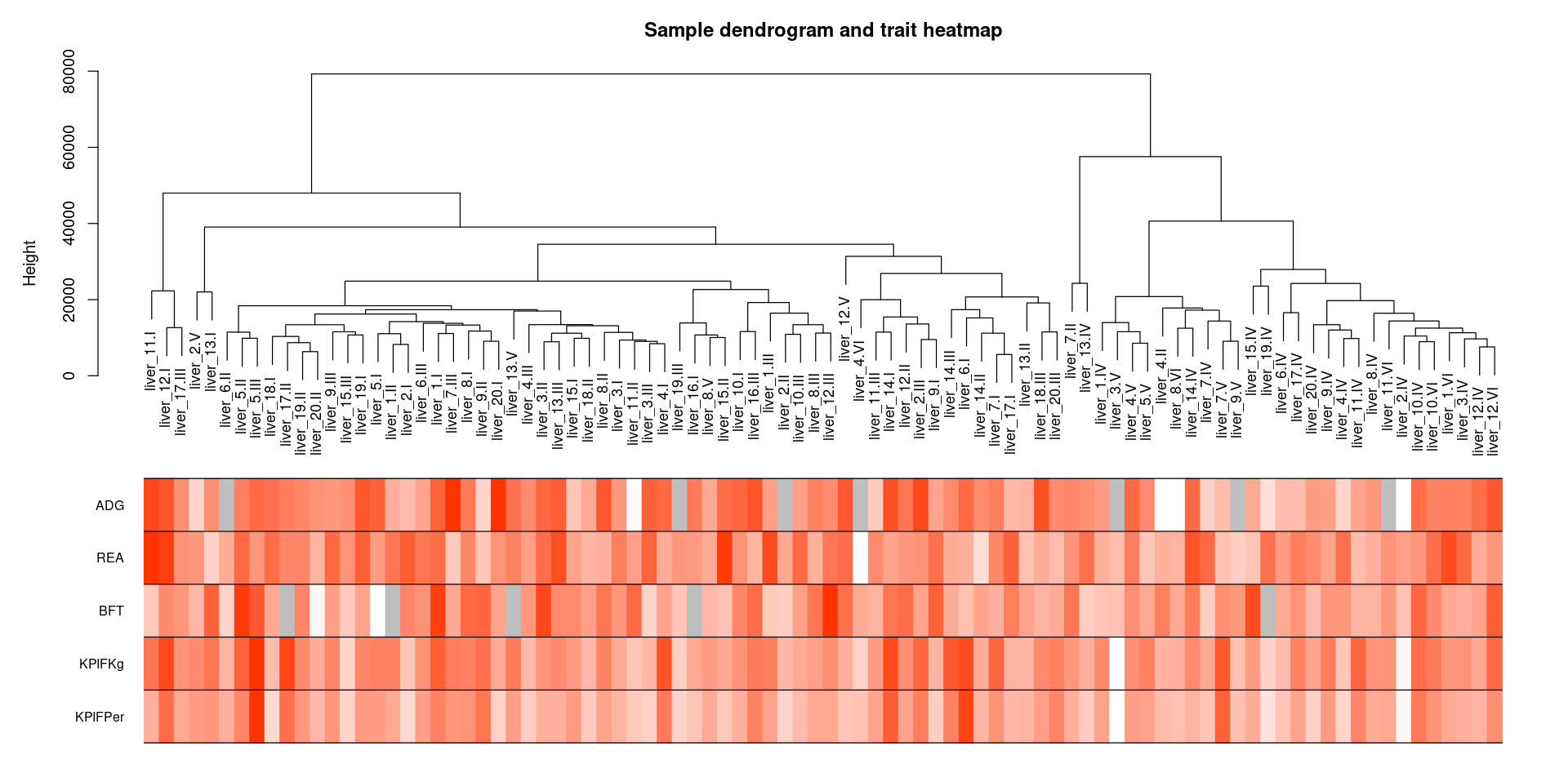


**Figure S7- Sample Dendrogram and Heatmap of carcass quality traits**

The dendrogram shows hierarchical clustering of individual samples based on gene expression profiles, while the heatmap below represents carcass quality traits for each sample. Traits include average daily gain (ADG), ribeye area (REA), backfat thickness (BFT), kidney, pelvic and inguinal fat in kilograms (KPIFKg), and as a percentage of carcass weight (KPIFPer). Color intensity indicates trait values, with darker red representing higher values and gray indicating missing data. This visualization was used to assess sample clustering and trait distribution before network analysis.


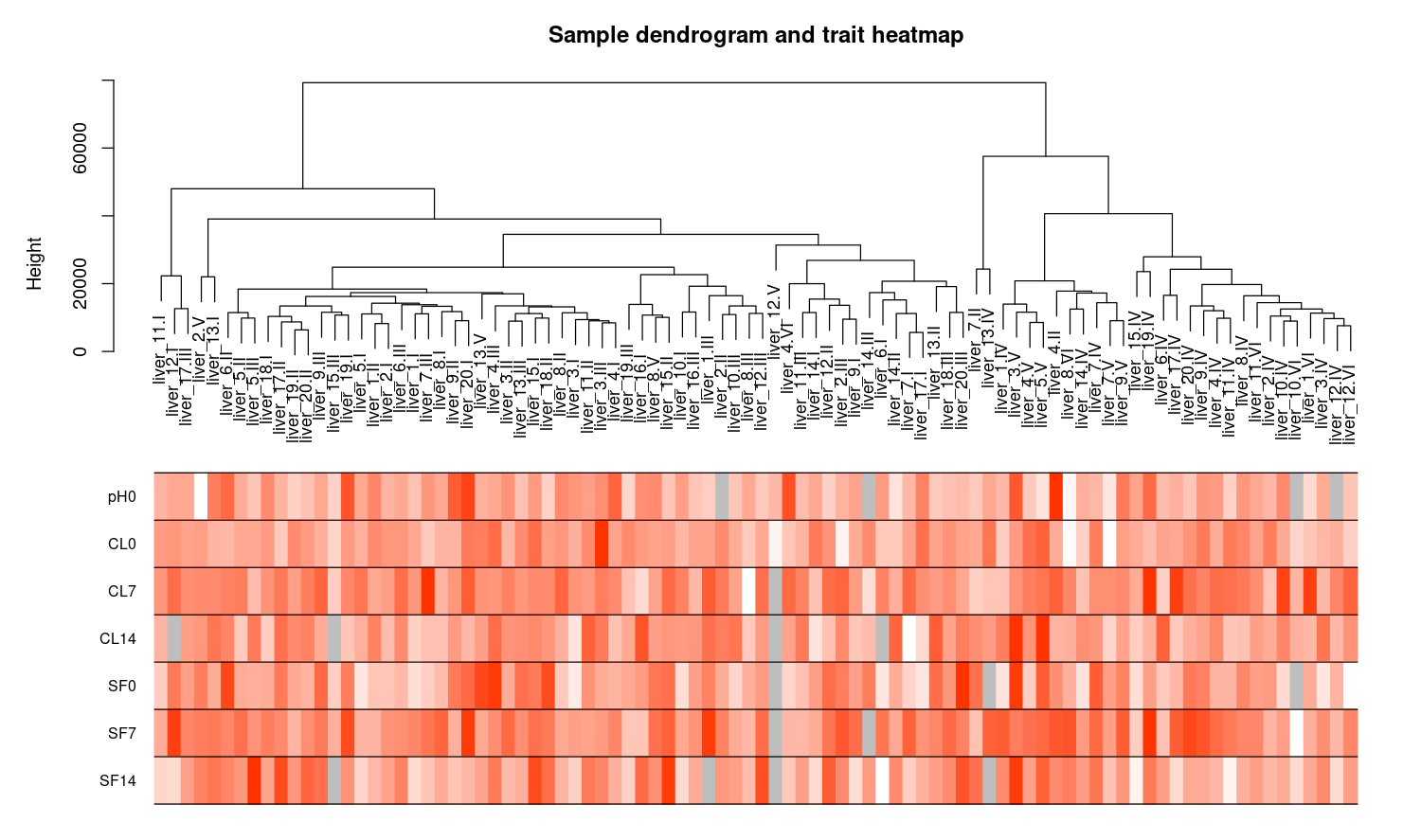


**Figure S8- Sample Dendrogram and Heatmap of meat quality traits**

The dendrogram shows hierarchical clustering of individual samples based on gene expression profiles, while the heatmap below represents meat quality traits for each sample. Traits include ultimate pH, cooking loss at 24 h postmortem (CL0), 7 days after slaughter (CL7), and 14 d after slaughter (CL14), and shear force measurements at 0, 7, and 14 d postmortem (SF0, SF7, SF14). Color intensity indicates trait values, with darker red representing higher values and gray indicating missing data. This visualization was used to assess sample clustering and trait distribution before network analysis.


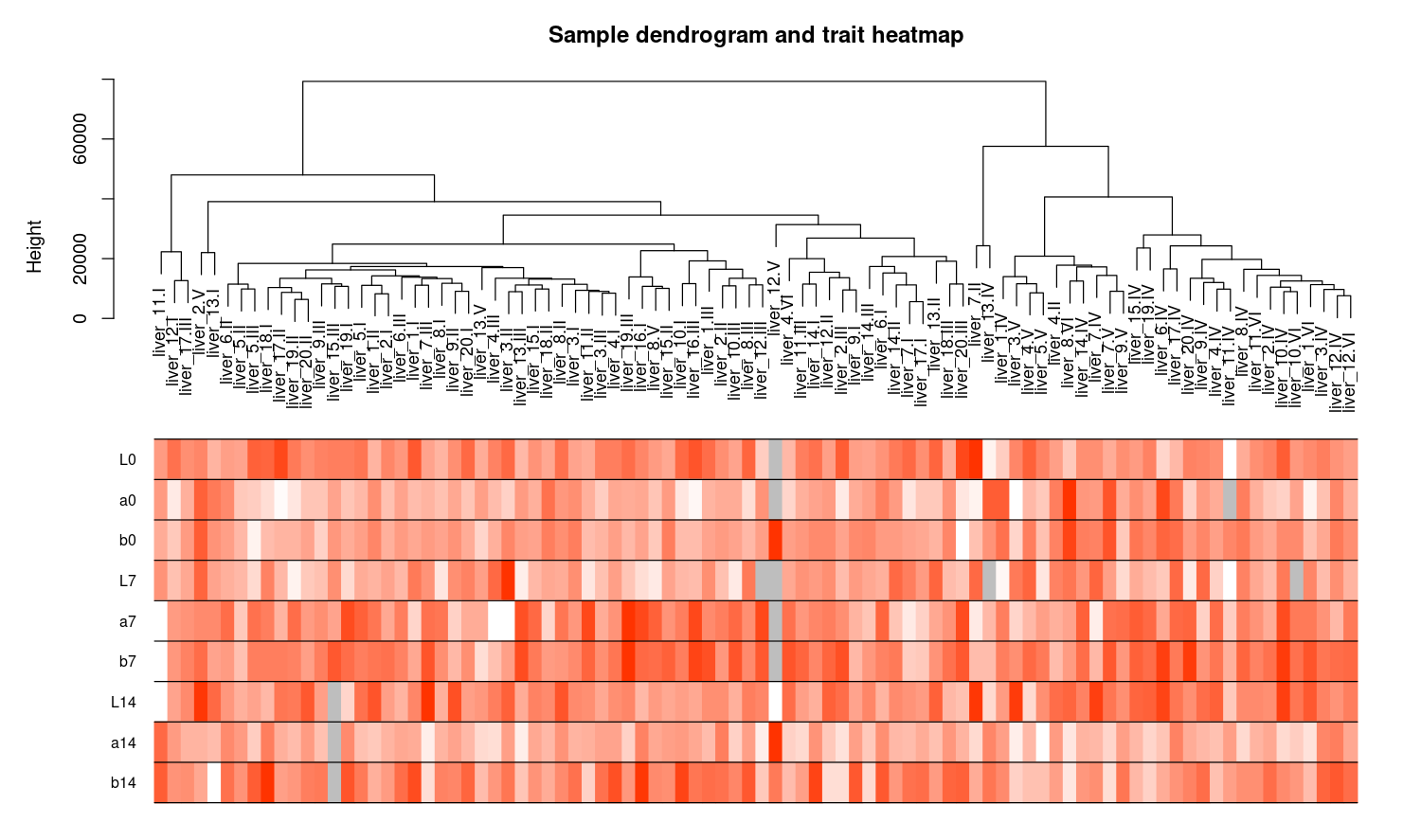


**Figure S9- Sample Dendrogram and Heatmap of Meat Color Traits**

The dendrogram shows hierarchical clustering of individual samples based on gene expression profiles, while the heatmap below represents meat color traits for each sample. Traits include lightness (L*), redness (a*), and yellowness (b*) measured at 0, 7, and 14 d postmortem (L*₀, a*₀, b*₀, L*₇, a*₇, b*₇; L*₁₄, a*₁₄, b*₁₄). Color intensity indicates trait values, with darker red representing higher values and gray indicating missing data. This visualization was used to assess sample clustering and trait distribution prior to network analysis.


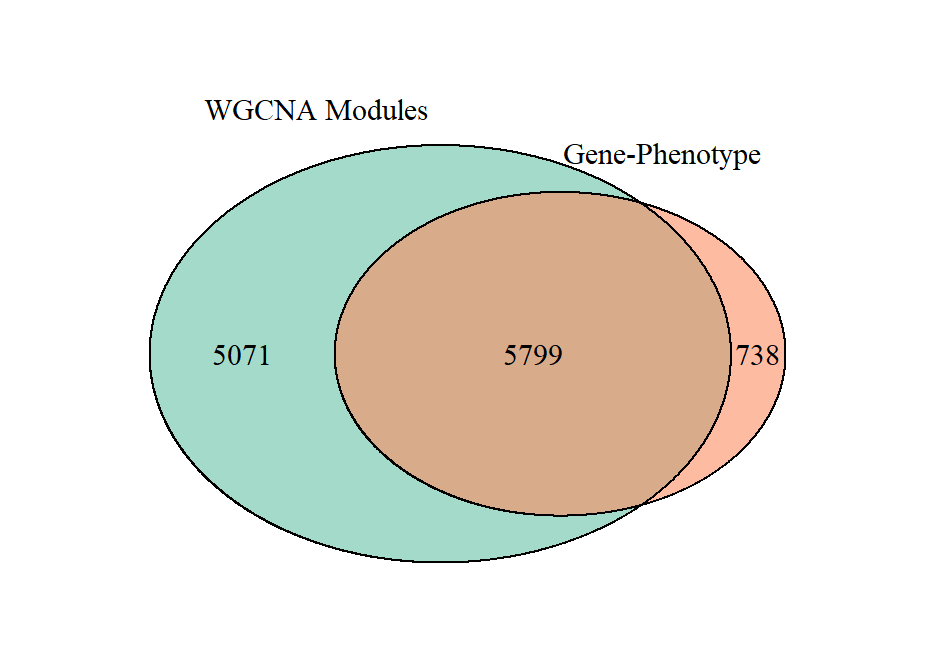


**Figure S10- Venn Diagram of the ten significant modules of WGCNA with the gene expression-phenotype analysis.**

The Venn diagram illustrates the intersection between genes identified in the ten significant WGCNA modules (green circle) and genes associated with phenotypes in the gene expression–phenotype analysis (orange circle). Numbers indicate the counts of genes unique to each set and those shared between the two analyses, highlighting common regulatory candidates.
